# miR-27b Represses Migration of Mouse MSCs to Burned Margins and Prolongs Wound Repair through Silencing SDF-1a

**DOI:** 10.1371/journal.pone.0068972

**Published:** 2013-07-22

**Authors:** Mu-Han Lü, Chang-Jiang Hu, Ling Chen, Xi Peng, Jian Chen, Jiong-Yu Hu, Miao Teng, Guang-Ping Liang

**Affiliations:** 1 Institute of Burn Research, Southwest Hospital, Third Military Medical University, Chongqing, P.R. China; 2 Department of Gastroenterology, Xinqiao Hospital, Third Military Medical University, Chongqing, P.R. China; The Chinese University of Hong Kong, China

## Abstract

**Background:**

Interactions between stromal cell-derived factor-1α (SDF-1α) and its cognate receptor CXCR4 are crucial for the recruitment of mesenchymal stem cells (MSCs) from bone marrow (BM) reservoirs to damaged tissues for repair during alarm situations. MicroRNAs are differentially expressed in stem cell niches, suggesting a specialized role in stem cell regulation. Here, we gain insight into the molecular mechanisms involved in regulating SDF-1α.

**Methods:**

MSCs from green fluorescent protein transgenic male mice were transfused to irradiated recipient female C57BL/6 mice, and skin burn model of bone marrow-chimeric mice were constructed. Six miRNAs with differential expression in burned murine skin tissue compared to normal skin tissue were identified using microarrays and bioinformatics. The expression of miR-27b and SDF-1α was examined in burned murine skin tissue using quantitative real-time PCR (qPCR) and immunohistochemistry (IHC), enzyme-linked immunosorbent assay (ELISA). The Correlation of miR-27b and SDF-1α expression was analyzed by Pearson analysis Correlation. miRNAs suppressed SDF-1α protein expression by binding directly to its 3′UTR using western blot and luciferase reporter assay. The importance of miRNAs in MSCs chemotaxis was further estimated by decreasing SDF-1α in vivo and in vitro.

**Results:**

miR-23a, miR-27a and miR-27b expression was significantly lower in the burned skin than in the normal skin (p<0.05). We also found that several miRNAs suppressed SDF-1α protein expression, while just miR-27a and miR-27b directly bound to the SDF-1α 3′UTR. Moreover, the forced over-expression of miR-27a and miR-27b significantly reduced the directional migration of mMSCs in vitro. However, only miR-27b in burn wound margins significantly inhibited the mobilization of MSCs to the epidermis.

**Conclusion:**

miR-27b may be a unique signature of the stem cell niche in burned mouse skin and can suppress the directional migration of mMSCs by targeting SDF-1α by binding directly to its 3′UTR.

## Introduction

Deep burns are one of the major challenges in the traditional treatment of burn injury, inhibiting tissue regeneration and wound repair [1]–[2]. The current trend in the healing of a deep burn is to apply tissue engineering for the recruitment and transdifferentiation of skin stem cells. However, these endogenous cells are extremely limited and cannot contribute to tissue repair effectively in large burn wound margins. The transplantation of mesenchymal stem cells is considered to play a critical role in the repair of damaged tissue [3]–[6], but it takes considerable time for recruited mesenchymal stem cells (MSCs) to reach their correct locations and assume their specific roles in wound healing. Yamaguchi et al. demonstrated that MSC-derived myofibroblasts appear at the upper dermis just beneath the regenerating epidermis mainly on postburn day 10 [7]. Verstappen et al. also found that fewer than 10% of MSC-derived cells contribute to palatal wound healing on postburn day 14 [8]. In our previous studies, MSC-derived epidermal cells and MSC-derived hair follicle cells appeared at burn wound margins but are not the main source of burn wound healing in the early stage of burns. However, MSCs do reach a peak on postburn day 28 in mice. In the phase of wound healing, the earlier the recruitment of MSCs to burn sites, the better.

Chemokines, as part of the cytokine network, are key factors regulating the movement of stem cells [9]–[10]. Currently, some signaling pathways including FAK,β-catenin, MMPs, FGF-4 et al. are closely governing MSCs migration, and stromal cell-derived factor-1 (SDF-1α, also named Cxcl12) is a powerful chemoattractant of both human and murine MSCs [11]–[12]. Interactions between SDF-1α and its cognate receptor CXCR4 [13] are crucial for the recruitment of MSCs from bone marrow (BM) reservoirs to damaged tissues and for repair during alarm situations [9]. Accumulating evidence shows that the increase of SDF-1α at the border zone of injury not only induces CXCR4-positive MSCs to the myocardial infarction area [14] but also recruits transplanted dermal multipotent stem cells to sites of injury in the skin [15]. The SDF-1α level reaches a peak on postburn day 7 in this study, but the increase of mMSCs to the wound margin becomes most significant during postburn days 14 to 21, and mMSCs decrease in the presence of CXCR4 inhibitor during the same time in our previous study (Date not shown). An increase in SDF-1α plays an important role in the chemotaxis, migration, and homing of MSCs after thermal injury, but this process is time-consuming. Therefore, enhancing endogenous SDF-1α release in the early stages of burn could be a clinically effective means of inducing MSC homing to the injured site. Although SDF-1α expression could be regulated by multiple transcription factors and various exogenous stimuli, the mechanism of the post-transcriptional regulation of SDF-1α is obscure.

MicroRNAs (miRNAs) are endogenous, small, non-coding RNAs that mediate gene expression by inhibiting the translation and inducing the degradation of target mRNAs by binding their 3′-untranslated regions (3′UTRs) [16]–[18]. Interestingly, homeostatic expression of SDF-1α is found in various normal skin cells, including fibroblasts, endothelial cells, and epidermal cells, while its expression increases after thermal injury. It is not clear whether SDF-1α-regulating miRNAs are down-regulated after burns. After discovering miRNAs that broadly regulate a variety of proteins, we hypothesized that miRNAs are likely involved in coordinating the expression of SDF-1α. If our hypothesis is correct, then tissues that differ in SDF-1α expression could be used in a comparative analysis to further identify differentially expressed miRNAs that may be responsible for controlling SDF-1α [19].

By in silico prediction, we detected 97 potential murine miRNA-binding sites within the SDF-1α 3′UTR. Then, using miRNA microarrays, we identified 85 miRNAs that were differentially expressed in burned murine skin compared to the bordering normal skin in our previous study. Five miRNAs overlapped between the bioinformatics and microarray analyses. Furthermore, miR-23a, which was detected by other researchers [20] but was not among the five miRNAs identified by our methods, was also included for comparative analysis. In this report, we sought to identify regulatory miRNAs that potentially target SDF-1α, which is involved in the release of MSCs in response to thermal injury, leading to MSC migration to the burned area. Finally, we sought to explore the mechanisms of functionally significant miRNA-mRNA interactions in burn wound healing.

## Materials and Methods

### Ethics

All animal experiments were approved by the Animal Care and Use Committee of the Third Military Medical University and were carried out in compliance with the ‘Guide for the Care and Use of Laboratory Animals’ published by the National Institutes of Health.

### miRNA Microarray Analysis

miRNA microarrays were applied to detect the miRNAs expression profiles of burned skin and the bordering normal skin tissue from the same mouse at postburn day 7 by Kang Chen Bio-tech (ShangHai, China). After measuring RNA concentration and quality on a NanoDrop, the samples were labeled using the miRCURY™ Hy3™/Hy5™ Power labeling kit and hybridized on the miRCURY™ LNA Array (v.11.0). Scanning was performed with the Axon GenePix 4000B microarray scanner. GenePix pro v.6.0 was used to read the raw intensity of the image. The capture probes for all miRNAs in all organisms were as annotated in Sanger miRBase Release 11.0 (http://microrna.sanger.ac.uk). A t-test was performed, and when the miRNA expression level changed at least ±2-fold with P<0.05, miRNA expression was considered significantly different.

### miRNA Target Gene Prediction

Computational analyses of miRNA-binding sites within the SDF-1α 3′UTR were carried out by TargetScan (http://www.targetscan.org/mmu_61/).

### MSCs Culture

**Figure 1:**
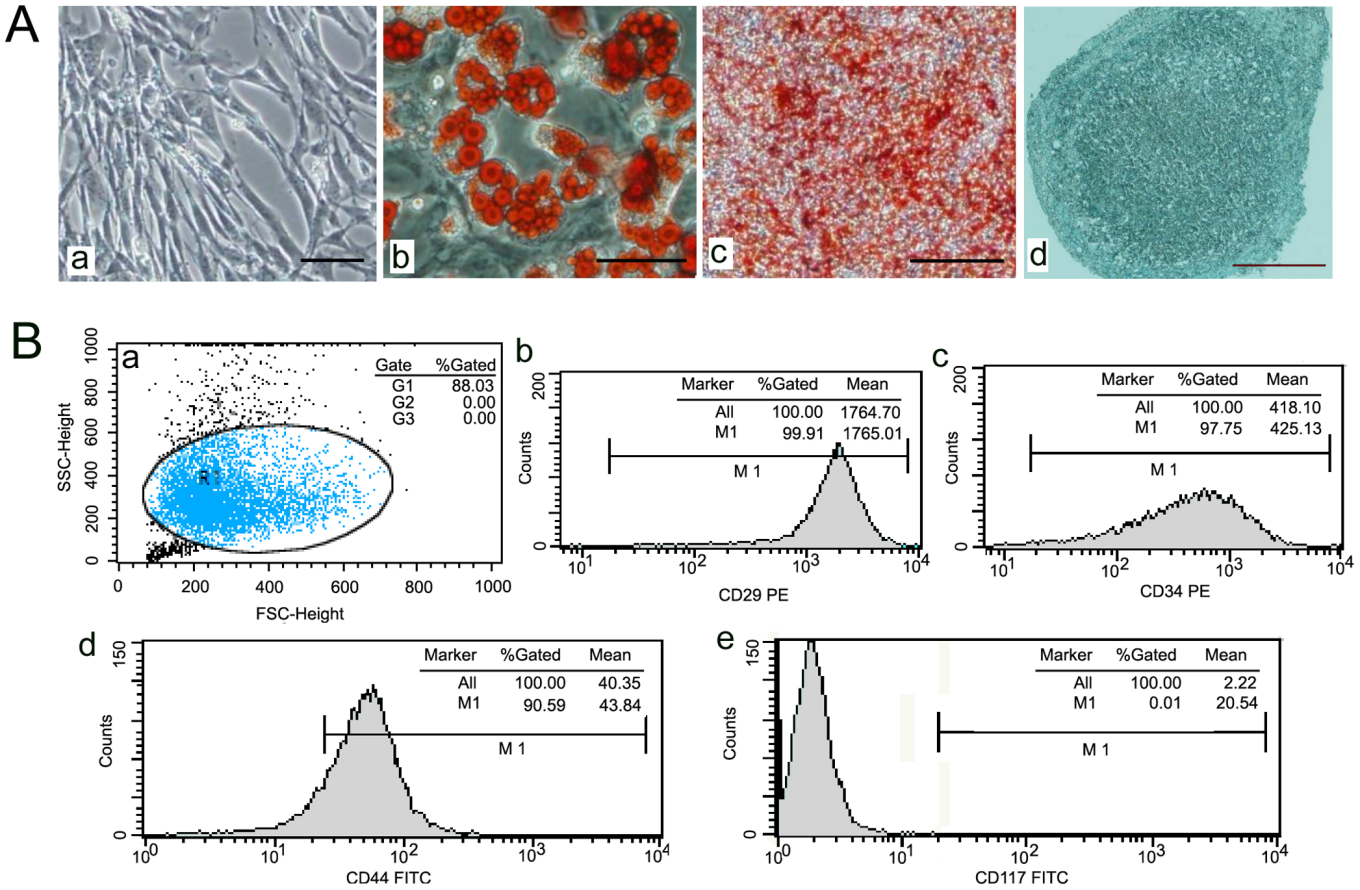
The identification of BM-MSCs. (A) The morphology of MSCs from male mice was observed under light microscopy. Bar, 100 um (a). mMSCs were induced to differentiate into adipocytes. Bar, 75 um (b), osteoblasts. Bar 400 um (c), or chondrocytes Bar, 75 um (d) using an in vitro differentiation assay. (B) The surface marker of MSCs were detected flow cytometry analysis(a), positive for CD29(b), CD34(c) and CD44(d), but negative for CD117 (e).

MSCs from male GFP-transgenetic C57BL/6 mice were purchased from Cyagen Biosciences (Cyagen Biosciences Inc., Guangzhou, China), and the morphology of the MSCs was spindle-shaped under a light microscope (Figure 1 Aa). The capacity of these MSCs to differentiate into adipocytes, chondrocytes, and osteoblasts was defined in vitro. (Figure 1 Ab, Ac, Ad) The surface marker of MSCs were detected flow cytometry analysis (Figure 1 Ba) and were positive for CD29, CD34 and CD44, but negative for CD117 (Figure 1 B b∼d). The MSCs were maintained in α-MEM (GIBCO, USA) supplemented with 10% fetal bovine serum (FBS; GIBCO, USA) at 37°C in a humidified atmosphere containing 5% CO_2_.

### Construction of Bone Marrow-chimeric Mice and Skin Burn Model

**Figure 2:**
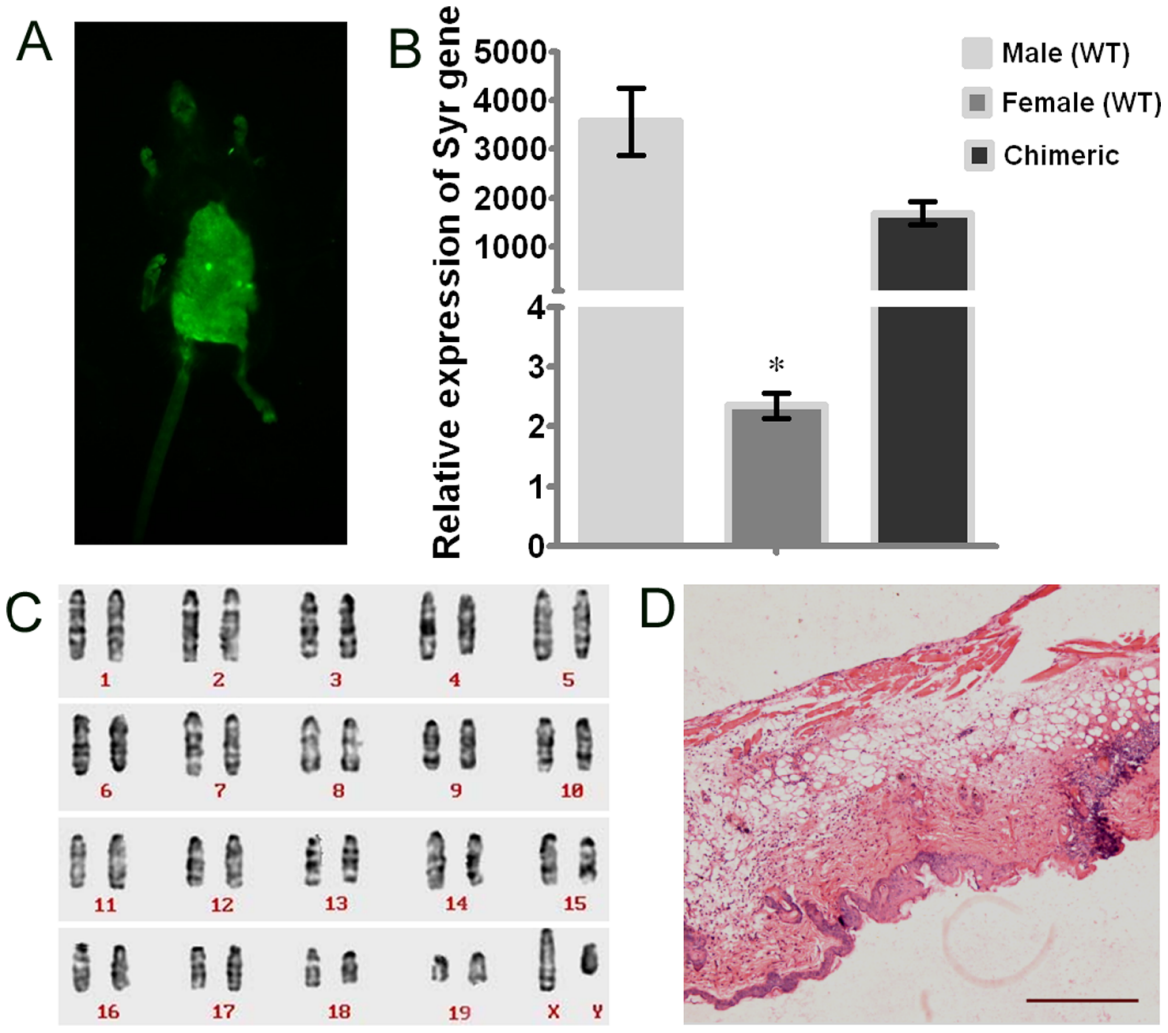
The identification of bone marrow-chimeric mice and skin burn model. (A) Chimeric mice was imaged under the in vivo imaging system. (B) Expression of the SRY gene in BM cells of different mice, assessed by qPCR. SRY gene expression was normalized to GAPDH (n = 3, *P<0.001 2-tailed Student’s paired t-test). The error bars represent S.D. (C) The chimeric model was confirmed by karyotype analysis. (D) HE staining shows deep burns on dorsal skin penetrating past the dermis. Bar, 400 um.

Female C57BL/6 mice were obtained from the Third Military Medical University, Chongqing, China. They were housed in autoclaved cages and treated with antibiotics for 10 days before irradiation and 2 weeks after irradiation. Recipient mice were treated with total-body ^60^Co γ-irradiation at a dose of 10 Gy at 0.985 Gy/min (Theratron-780 model, MDS Nordion, Ottawa, ON, Canada) and four hours after irradiation, infused with a dose of 1×10^6^ MSCs originating from male GFP-transgenic C57BL/6 mice via the tail vein to reconstitute the females’ hematopoietic system. The green fluorescence of the bone marrow-chimeric mice were imaged under the in vivo imaging system (Figure 2 A). The chimeric mice were further identified by detecting the level of Sry protein in peripheral blood and by karyotype analysis (Figure 2 B, C), and they were bred in specific pathogen-free conditions for 4 weeks. Round aluminum templates heated to 100°C and applied for 8 s to the moistened and depilated dorsal skin produced a circular spot of 1.5 cm diameter on the mice [21]. The desired depth of injury was confirmed by HE staining (Figure 2 D).

### SDF-1α in the Damaged Skin Tissue Identified by Immunohistochemistry (IHC)

Burned skins were harvested in 4% PBS-buffered formaldehyde, embedded in paraffin and then sliced into sections 4 µm thick. The SDF-1α (Abcam, UK) antibody was used for immunohistochemical analysis. Immunoreactivity in sections was detected using a horseradish peroxidase-3,3′-diaminobenzidine kit (Beyotime, China), followed by counterstaining with hematoxylin, dehydration and mounting. Five areas containing SDF-1α-positive cells were microscopically and quantitatively analyzed [22].

### Quantification of miRNAs by Quantitative Real-time PCR (qPCR)

Total RNA was isolated from burned skin tissue of bone marrow-chimeric mice (after 7 days of thermal injury) and the surrounding normal skin tissue using RNAiso plus (TAKARA, Japan), and cDNA was subsequently PCR-amplified accordingly to the methods described as previously [24]. U6 was used as the endogenous control. The gene-specific primer pairs used to amplify specific target genes were as follows, and GenBank accession numbers also be included: mmu-miR-1(NR_029528.1): GSP, 5′-GGGGTGGAATGTAAAGAAGT-3′ and reverse, 5′-CAGTGCGTGTCGTGGAGT-3′; mmu-miR-136(AJ 459747.1): GSP, 5′-GGAACTCCATTTGTTTTGA-3′ and reverse, 5′-CAGTGCGTGTCGTGGAGT-3′; mmu-miR-214(NR_029796.1): GSP, 5′-GACAGCAGGCACAGACA-3′ and reverse, 5′-TGCGTGTCGTGGAGTC-3′; mmu-miR-23a (NR_029740.1): GSP, 5′-CCATCACATGCCAGG-3′ and reverse, 5′-CAGTGCGTGTCGTGGAGT-3′; mmu-miR-27a (NR_029746.1): GSP, 5′-GGGGTTCACAGTGGCTAA-3′ and reverse, 5′-CAGTGCGTGTCGTGGAGT-3′; mmu-miR-27b(NR_029531.1): GSP, 5′-GGGGTTCACAGTGGCTAAG′ -3′ and reverse, 5′-CAGTGCGTGTCGTGGAGT-3′; U6(NM_001204274.1): forward, 5′-GCTTCGGCAGCACATATACTAAAAT-3′ and reverse, 5′-CGCTTCACGAATTTGCGTGTCAT-3′; VEGF (NC_000083.6): forward, 5′-GTCCAACTTCTGGGCTCTTCT-3′ and reverse, 5′-CCTTCTCTTCCCCTCTCT-3′.

### Design of Lentiviral Construct and Infection

The lentiviral vectors, obtained from GeneChem Management Inc. (Shanghai, China), contained pre-miRNAs and GFP. They were named LV-mmu-mir-1, LV-mmu-mir-136, LV-mmu-mir-214, LV-mmu-mir-23a, LV-mmu-mir-27a, LV-mmu-mir-27b, and LV-cel-mir-67 (the negative control). mMSCs were infected at a multiplicity of infection (MOI) of 10 and sorted by a FACSAria Cell Sorter (BD, USA) using laser excitation at 488 nm after infection for 96 hours. The percentage of positive cells (green cells) was nearly 100%, as evaluated by laser scanning confocal microscopy (Leica, Germany) [24].

### 3-(4,5-Dimethylthiazol-2-yl)-2,5-diphenyltetrazolium Bromide (MTT) Assay

The assay was tested to calculate the inhibition rate of MSC viability treated by LV-mmu-mir-27b, and LV-cel-mir-67. After the The cell lysates were collected, 12.5 µM MTT dye was added to each well, and the cells were further incubated at 37°C for 4 h. The Optical density values of the absorbance 490 nm were obtained using a microplate reader (mode FL 330, Bio-Tek Instruments, Winooski, VT, USA).

### Enzyme-linked Immunosorbent Assay (ELISA)

The concentration of SDF-1α was measured in the skin tissue of C57BL/6 mice on postburn days 0, 1, 3, 7, 14, 21, and 28 using ELISA (RayBiotech, USA). SDF-1α protein was quantified using a DENLEY DRAGON Wellscan MK3 instrument (Thermo, USA) [23]. The concentration of IL-8 and Pan-CK were measured in the MSCs over-expressing cel-miR-67 and miR-27b using ELISA (RayBiotech, USA). IL-8 and Pan-CK protein was quantified using a DENLEY DRAGON Wellscan MK3 instrument (Thermo, USA) [23].

### Western Blot

Protein was extracted and separated by SDS-PAGE and transferred to polyvinylidene difluoride membranes (Millipore, USA). Following blocking of non-specific protein, membranes were incubated with antibodies against SDF-1α (1∶500, eBioscience, USA) and GAPDH (1∶3000, Santa Cruz, USA). Horseradish peroxidase-conjugated secondary antibodies (ZhongShan, China) were diluted 1∶5000. The results were visualized using enhanced chemiluminescence (ECL, Thermo, USA), and densitometry was assessed with a Gel-DOC 2000 imaging scanner (BIO-RAD, USA) [25].

### Luciferase Reporter Assay

The SDF-1α 3′UTR was amplified from murine cDNA and cloned into the DraI and XbaI restriction sites of the pMIR-REPORT vector (Promega, USA). Putative miRNA binding sites in the pMIR-SDF-1α 3′UTR were mutated using the QuikChange Site-Directed Mutagenesis Kit (Stratagene, USA). HEK293 cells were co-transfected with 100 ng of luciferase plasmid and 100 nM miRNA mimic or control mimic (RIBOBIO, China) in 96-well plates using Lipofectamine 2000 Reagent (Invitrogen, USA). After 24 h transfection, Fireﬂy and Renilla luciferase activities were assayed using the Dual-Luciferase Reporter Assay (Promega, USA) [25].

### Cell Chemotaxis Assay in vitro

MSCs migration was measured using a Transwell Chamber assay (Corning, USA). MSCs infected with lentivirus were trypsinized and resuspended in α-MEM containing 10% (v/v) FBS at 1×10^5^ cells/ml, and a 600 µl cell suspension was added to the lower well. Normal MSCs were trypsinized and resuspended in α-MEM containing 0.1% (v/v) FBS at 5×10^5^ cells/ml, and a 100 µl cell suspension was added to the upper chamber. After 24 h incubation, cells were swabbed away from the inside of the chamber, and cells adhering to the underside of the chamber were fixed for 30 min in 4% paraformaldehyde. Cells were stained with 0.5% (w/v) crystal violet (Beyotime, China) and counted in five random fields under light microscope at 100 × magnification (Olympus, Japan) [24].

### Fluorescence in situ Hybridization (FISH) and Immunofluorescence (IF) Analysis of MSCs Chemotaxis in vivo

The burn-injured chimeric mice were generated as described in the above section. Then, 0.1 ml LV-mmu-mir-27b (5×10^8^ TU/ml) was added to the burn wound margins in each of two separate multi-point injections. LV-cel-mir-67 was injected as negative control and PBS as blank control in the same way. The skin tissues were removed and cut into frozen sections at postburn day 21. Sections were incubated with the primary monoclonal Pan-CK antibody (1∶50 Santa Cruz, USA) at 4°C overnight. DNA of the Y chromosome was denatured, and the hybridization process was performed on the next day following the manufacturer’s protocol (starFISH Cambio, England). The break-apart probe set included DNA fragments against the Y chromosome labeled with Cy3 (Cambio, England). On the third day, the slides were washed to clear unbound DNA sequences, followed by incubated in primary monoclonal Pan-CK antibody (1∶50) at 37°C for 5 min and in secondary antibody (1∶200) labeled with FITC (Invitrogen, USA) at 37°C for 90 min, and counterstaining nuclei with DAPI (Beyotime, China) [26]. The sections were analyzed by laser scanning confocal microscopy in ten random fields (Leica, Germany). Experiments were performed in triplicates. Standardized images of wounds were recorded using a digital camera to analyze wound sizes daily.

### In vivo ﬂuorescence Imaging

In vivo ﬂuorescence imaging was performed everyday post wounding using a Maestro In Vivo Imaging System(Cambridge Research & Instrumentation, Boston, MA). The animals were placed on the microscope stage, The measurements of the burn area were analyzed using the Maestro 2.10.0 software, which of the wound area were taken three times by two independent experimenters.

### Statistical Analysis

The data are presented as the mean ± S.D. Significance between groups was determined with the Mann-Whitney test or Wilcoxon matched-pair test. A value of P<0.05 was considered statistically significant. The analyses were carried out with Prism 5.0 (GraphPad).

## Results

### Six Down-regulated miRNAs were Identified as SDF-1α-regulating miRNAs by Microarray and Bioinformatics Analyses

**Figure 3:**
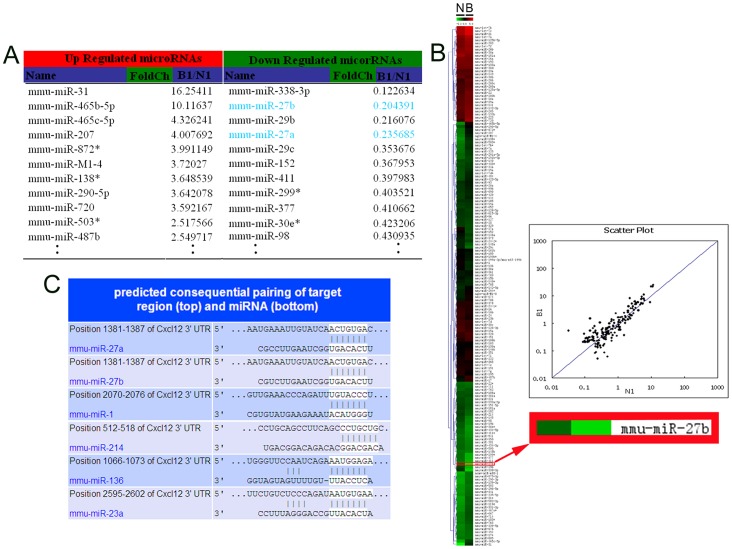
Microarray-based miRNA expression profiling and bioinformatics. (A) The list of miRNAs whose expression was altered in burned murine skin compared to the bordering normal skin. The threshold value used to screen upregulated and downregulated miRNAs with fold change ≥1.5. (B) The heat map shows the result of the two-way hierarchical clustering of genes and samples. Each row represents a miRNA, and each column represents a sample (N: normal skin; B: burned murine skin). The color scale shown at the top illustrates the relative expression level of a miRNA: red represents high expression, green represents low expression. miR-27b was downregulated significantly in burned murine skin compared to the bordering normal skin. The scatter plot’s correlation R-value for the two samples reflects the reproducibility of the slides. (C) A putative target site of miRNAs highly conserved in the SDF-1α mRNA 3′UTR as predicted by bioinformatics analysis.

First, we identified SDF-1α-targeting miRNAs as predicted by TargetScan (http://www.targetscan.org/mmu_61/). It predicted 97 miRNAs that possibly suppressed SDF-1α expression post-transcriptionally by binding to the SDF-1α 3′UTR. The miRNA microarray showed that 85 miRNAs’ expression levels were changed in burned murine skin compared to the bordering normal skin significantly (Figure 3A, B and the GEO accession number is GSE46660, http://www.ncbi.nlm.nih.gov/geo/query/acc.cgi?acc=GSM1133639). Five miRNAs overlapped between the two results (Figure 3C). However, miR-23a, which has been identified to directly regulate SDF-1α 3′UTR by other researchers, was not among the five miRNAs identified by our methods.

### Expression of SDF-1α and SDF-1α-regulating miRNAs in Burned Murine Skin Tissue

**Figure 4:**
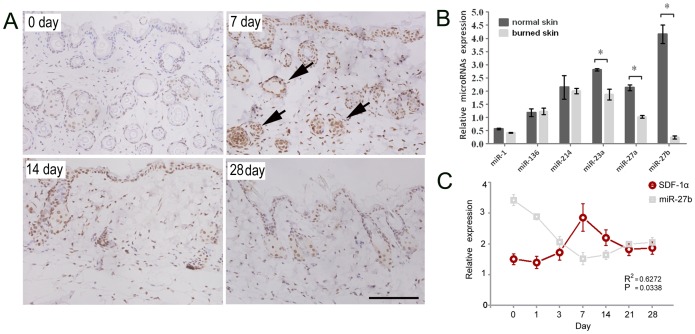
The expressions of SDF-1α and different miRNAs in the burned skin of mice. (A) The expression of SDF-1α in damaged skin tissue at different time points, as measured by IHC. SDF-1α was continuously expressed in the basal layer of the epidermis and surrounding hair follicles and reached a peak at the postburn day 7. The arrows show that the majority of SDF-1α was located in the stroma cells of the dermis, epidermal cells of the burned margin, and sebaceous gland cells of the dermis. Bar, 100 µm. (B) The expression of related miRNAs in the damaged skin tissue at postburn day 7, as measured by qPCR. (n = 3, *p<0.05; Wilcoxon marched-pair test). The error bars represent S.D. (C) SDF-1α protein level in the tissue of burned mice was measured by ELISA at different time points after thermal injury. The concentration of SDF-1α increased slowly in damaged skin tissues and reached a peak at postburn day 7 (n = 3, *P<0.05 compared to day 0, Mann-Whitney test). The error bars represent S.D. mir-27b level in the same tissue of burned mice was measured by qPCR(n = 3, *p<0.05; Wilcoxon marched-pair test). The error bars represent S.D. The correlation between mir-27b and SDF-1α were tested by Pearson analysis. (R^2^ = 0.6272, P<0.05).

A deep burn was induced in bone marrow-chimeric mice by thermal injury to the skin. The mice were sacrificed for skin removal on different postburn days to allow SDF-1α detection by IHC analysis. It showed that SDF-1α was continuously expressed in the basal layer of the epidermis and surrounding hair follicles; during wound healing, the majority of SDF-1α was located in the stromal cells of the dermis, epidermal cells of the burned margin, and sebaceous gland cells of the dermis. The expression of SDF-1α reached a peak at postburn day 7 (Figure 4A). The down-regulation of SDF-1α-regulating miRNAs in burned skin tissue (from chimeric mice at postburn day 7) compared to normal surrounding tissue was also confirmed by qPCR. The expression of miR-23a, miR-27a and miR-27b were significantly lower in the burned skin compared to the normal skin (p<0.05) (Figure 4B). Then, ELISA showed that the concentration of SDF-1α expression increased slowly in burned skin tissues and reached a peak on postburn day 7 (p<0.05) (Figure 4 C). Meanwhile, miR-27b was found negative related to the change of SDF-1α, (P<0.05) in Pearson test by Prism 5.0 (GraphPad) (Figure 4 C). These results suggest that miR-23a, miR-27a, and miR-27b might be involved in the regulation of SDF-1α in the context of burned skin.

### Identification of SDF-1α-regulating miRNAs by Western Blot and Luciferase Assays

**Figure 5:**
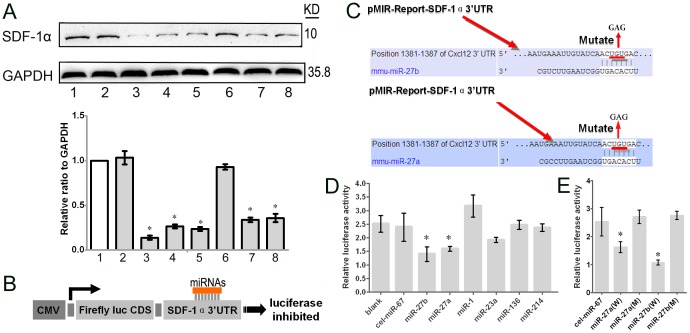
The effect of the miRNAs on the level of SDF-1α and luciferase of the 3′UTR. (A) The expression of SDF-1α protein in MSCs after 96-hour infection with lentiviral vectors expressing the given miRNA, detected by western blot. Lane 1, MSCs; lane 2, MSCs/cel-miR-67; lane 3, MSCs/miR-27b; lane 4, MSCs/miR-27a; lane 5, MSCs/miR-1; lane 6, MSCs/miR-23a; lane 7, MSCs/miR-136; lane 8, MSCs/miR-214. The relative expression ratio to GAPDH was summarized in the below panel. (B) The amplified wild-type SDF-1α 3′UTR was cloned into the dual-luciferase miRNA target expression vector, and potential binding sites for miRNAs in the 3′UTR of SDF-1α were predicted by computational analysis. (C) To test for potential interactions with miRNAs, the SDF-1α 3′UTR and the given miRNA were co-transfected into HEK293 cells. The activities of Fireﬂy and Renilla luciferase were assayed 24 h post-transfection (n = 3, *p<0.05; Mann-Whitney test). The error bars represent S.D. (D) The predicted miR-27a and miR-27b binding sites in SDF-1α were mutated by site-directed mutagenesis of the miRNA seed region. (E) The Fireﬂy and Renilla luciferase activities were analyzed in HEK293 cells 24 hours after co-transfection with the given miRNA and the mutated SDF-1α 3′UTR. (n = 3, *p<0.05; Mann-Whitney test). The error bars represent S.D.

To examine whether the above miRNAs could directly regulate SDF-1α expression, we performed western blots to investigate SDF-1α expression in mMSCs that had been infected with different miRNA lentiviruses for 96 hours. As shown in Figure 5A, miR-27b, miR-27a, miR-1, miR-136, and miR-214 reduced the level of SDF-1α protein. In particular, miR-27b suppressed SDF-1α protein in mMSCs most significantly compared to the control group (Figure 5A). To confirm the underlying mechanism by which these miRNAs reduce the expression of SDF-1α, we investigated their SDF-1α 3′UTR binding sites and found that they all had separate binding sites (Figure 5B). miR-27a and miR-27b repressed the luciferase activity of the pMIR-Report-SDF-1α 3′UTR compared to the negative control and blank control, while other miRNAs did not (Figure 5C). As expected, miR-27a- and miR-27b-mediated suppression of the Fireﬂy/Renilla luciferase activity was abolished when we mutated the miR-27a and miR-27b binding sites in the SDF-1α-3′UTR (Figure 5D, E), suggesting that miR-27a and miR-27b inhibit SDF-1α translation by directly binding to the SDF-1α 3′UTR.

### miR-27b Inhibits mMSC Directional Migration and Wound Healing Ability

**Figure 6:**
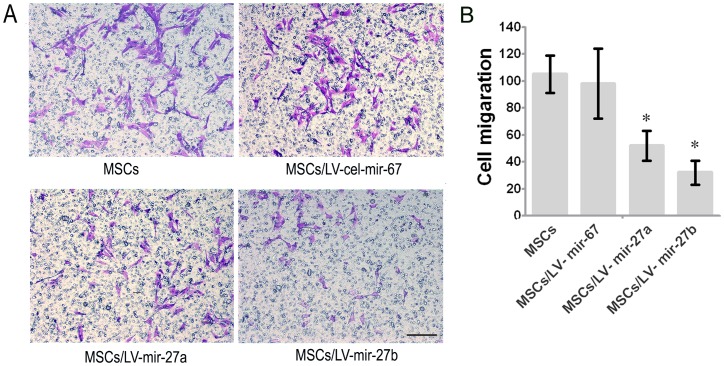
The effects of miR-27a and miR-27b on MSC migration. (A) The migration capacity of MSCs over-expressing miR-27a and miR-27b was analyzed with a transwell migration assay compared to the blank control and negative control. Bar, 100 um (B) Alteration of the chemotactic capacity of MSCs in different niches (n = 5, *p<0.05; Mann-Whitney test). The error bars represent S.D.

**Figure 7:**
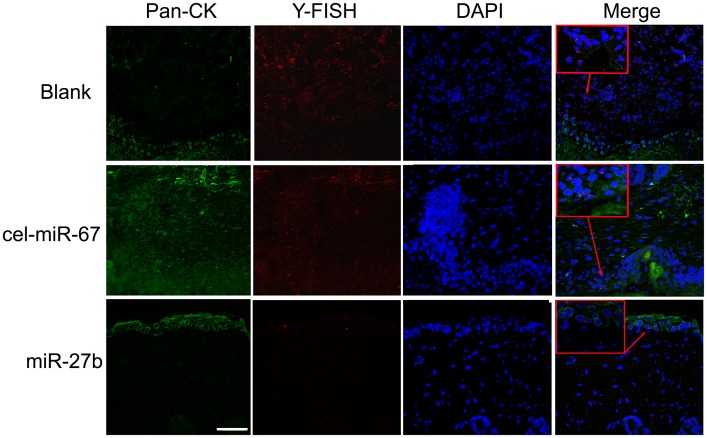
Inhibition of SDF-1α attenuates mobilization. The LV-mmu-mir-27b treatment inhibited the mobilization of mMSCs to the epidermis. Pan-CK (green), Y chromosome (red) and DAPI (blue). Bar, 50 µm.

**Figure 8:**
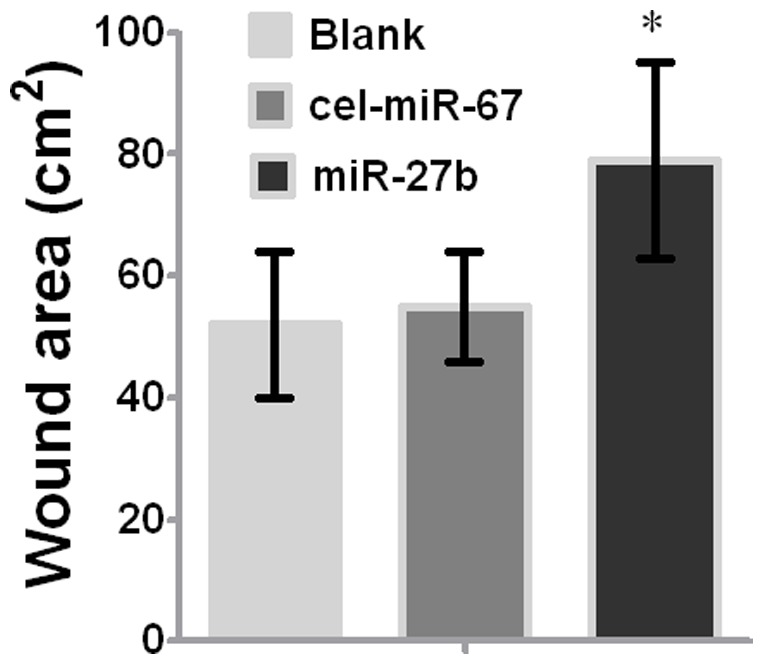
Wound healing of mMSCs. Wound size was measured at postburn day 21 after treating wounded mice with LV-mmu-mir-27b or PBS (n = 6; *, P<0.05, Mann-Whitney test). The error bars represent S.D.

The above results clearly demonstrate that miR-27a and miR-27b greatly suppressed the expression of SDF-1α. We speculated that miR-27a and miR-27b may function as chemotaxis inhibitors in burn wound healing. First, we overexpressed specific miRNAs in mMSCs to reduce SDF-1α secretion and found that miR-1 (see Figure 6 and Figure S1), miR-27a and miR-27b significantly inhibited the migration of other normal mMSCs compared to negative controls in vitro (p<0.05) (Figure 6). Next, FISH and IHC were used to measure the amount of MSC chemotaxis to the burn wound sites after the injection of LV-mmu-mir-27a or LV-mmu-mir-27b into the burn wound sites of bone marrow-chimeric mice. Y-chromosome and Pan-CK double-positive epidermal cells and hair follicle cells at the wound margins were decreased in the LV-mmu-mir-27b group (Figure 7). These results show that miR-27b functionally down-regulated SDF-1α expression and eventually inhibited the migration of mMSCs to burn wound niches. Meanwhile, wound size was measured every day after treatment. As shown in Figure 8, treatment with LV-mmu-mir-27b significantly prolonged wound closure compared to the negative control (cel-miR-67) and blank control. These results demonstrate the importance of the interaction between miR-27b and SDF-1α in regulating the migration of mMSCs in wound healing.

## Discussion

SDF-1α promotes CXCR4-positive MSC egress from the stem cell pool, which then home to damaged sites along the gradient of SDF-1α expression. SDF-1α expression is dynamic and could define the variable stem cell niche during physical stress [9], [27]. Interestingly, the increase of SDF-1α level is not obvious in the early stage of burns.

Little is known as to how distinct functional compartments are specified within this niche of MSCs. Accumulating evidence suggests that miRNAs affect the stem cell self-renewal, differentiation, and chemotaxis. Moreover, some miRNAs are differentially expressed in niches of stem cells [12], [28]–[30], suggesting a specialized role in stem cell regulation [31]. Herein, to gain insight into the molecular mechanisms involved in regulating SDF-1α, we focus on its regulation by miRNAs. Identifying the target mRNAs of miRNAs is a first step to explore the function of the miRNAs. The down-regulation of miRNAs was in response to simultaneous induction of SDF-1α expression in thermal injury. The prevention of SDF-1α degradation by the elimination of certain miRNAs might increase the mobilization of CXCR4-positive and SDF-1-responsive MSCs from BM to the wound sites.

miRNAs negatively regulate target gene expression by binding to the 3′UTR of mRNAs by either initiating degradation of the mRNA or preventing its translation to protein [32]–[33]. Therefore, we performed luciferase reporter assays to determine whether miR-27a and miR-27b are capable of binding the 3′UTR of SDF-1α to prevent its translation. Western blot analysis demonstrated that miR-27b caused a significant reduction of SDF-1α (p<0.05), while other miRNAs had minor or no effects on SDF-1α translation (Figure 5A), which did not confirm the results of the luciferase reporter assay. Thus, we performed mutation assays of the putative binding sites to confirm that the SDF-1α 3′UTR is a direct target of miR-27a and miR-27b (Figure 5 E). These results illustrate the limitations of current bioinformatic prediction for discerning miRNA-mRNA interactions of potential significance [34]. In our previous studies, mMSCs were identified as a major source of SDF-1α and its cognate receptor, although SDF-1α is secreted by diverse cell types [24]. Therefore, we used a migration assay to confirm that SDF-1α-regulating miRNAs are able to break the autocrine loop of reciprocal modulations in the SDF-1α/CXCR4 axis and inhibit the homing of other normal MSCs (Figure 6A, B). To further confirm the effect of miRNA-27b on the migration and recruitment of MSCs but not the other aspects of cell behaviors, we tested the proliferation, angiogenesis and the differentiation of the MSCs. The results showed that miRNA-27b did not affect MSCs proliferation, the ability of secretion of IL-8 which is able to induce angiogenesis and cell movement, differentiation of MSCs through testing Pan-CK. However, miRNA-27b could inhibit the VEGF mRNA, which suggested that miRNA-27b might inhibit the angiogenesis of the burn area (Figure S2). Altogether, these results further indicate the importance of the miRNA-27b-SDF-1α-mediated inhibition of MSCs migration in the wound repair.

Interestingly, miR-1, miR136 and miR214 inhibited SDF-1α protein expression without affecting the luciferase activity of the p-MIR-Report-SDF-1α 3′UTR. Because 47% of miRNA target sequences are located in the 3′UTRs of mRNAs, 47% of them in open reading frames (ORFs) and the rest in the 5′UTR [35]–[36], we speculate that miR-1, miR136 and miR214 may bind to the ORF or 5′UTR of SDF-1α, but confirmation require further investigation. Interestingly, miR-214 reduced SDF-1α expression but did not inhibit the directional migration of MSCs (Figure S1). Overexpression of miR-1 in hypertrophic cardiomyocytes suppresses cytoskeleton regulatory protein twinfilin-1 to reduce the cell size and attenuate the expression of hypertrophic markers [37]. miR-214 is a post-transcriptional regulator of AP-2α, a transcription factor (TF) that inhibits the cancer cell metastatic phenotype. miRNAs may predominate among TF targets, suggesting that the two classes of molecule could be connected in regulatory networks. TF–miRNA interactions may function together in composite feedback loops that consist of mutual regulation at a global level [38].

Although in vitro experimentation enables manipulations in a more simple model, it might oversimplify the physiologic stress conditions. We therefore showed that forced overexpression of miR-27b on burn wound margins significantly inhibited the mobilization of MSCs to the epidermis (Figure 7) and wound closure (Figure 8), while miR-1 and miR-27a had no obvious effects on the homing of MSCs in vivo. These findings suggest the combined action of multiple regulators in vivo will determine the final trafficking of MSCs to the skin wound by accelerating SDF-1α-induced migration and differentiation into epidermal cells. These results also contribute to our understanding that a single specific miRNA or miRNA group interaction play a decisive role, and the miRNAs interaction effect is synergistic or still some competition in the special microenvironment. At the post-transcriptional level, miR-27b serves as a biological rheostat [39] whose function depends on the mutual/reciprocal actions of the ligand SDF-1α and its receptor CXCR4 expressed by stem cells and stromal cells under different alarm situations.

In addition, the nonmonogamous relationships between SDF-1α and miRNAs, which also include miR-126 and miR-886-3p (as predicted by other bioinformatics algorithms), regulate the expression of SDF-1α [19], [22], further complicating the interplay between these miRNAs and its target gene. However, these miRNAs were not expressed differentially between the burned skin and surrounding normal skin in this study. Beyond the findings that miRNAs may be unique signatures of different stem cell niches, our findings also suggest that antagonistic action may exist within the 3′UTR [36], possibly as a result of conformational changes occurring upon miR-27b binding. The underlying mechanism by which miRNAs affect SDF-1α requires further investigation.

In conclusion, our results have demonstrated that miR-27b suppressed MSC directional migration by down-regulating SDF-1α expression. It is probable that the miR-27b-mediated down-regulation of SDF-1α in the early postburn stage delayed the wound repair, and the inhibition of miR-27b may upregulate SDF-1α protein expression and ultimately promotes MSC directional migration and wound healing. In addition, we are currently evaluating the possibility that miR-27b is a potential prognostic indicator in burn wound healing.

## Supporting Information

Figure S1 **The effects of miR-23a, miR-136, miR-1 and miR-214 on MSC migration.** (A) The migration capacity of MSCs over-expressing miR-23a, miR-136, miR-1 or miR-214 in a transwell migration assay was compared to the blank control and negative control. Bar, 100 um (B) Alteration of the chemotactic capacity of MSCs in different niches (n = 3, *p<0.05; independent-sample t test). The error bars represent S.D.
(TIF)

Figure S2 **The effects of miR-27b on MSCs behavior.** (A) The proliferation of MSCs over-expressing miR-27b in MTT assay was compared to the negative control.(B)The effects of miR-27b on VEGF in qPCR was compared to the negative control.(C) The effects of miR-27b on IL-8 and Pan-CK in ELISA was compared to the negative control.(n = 3, *p<0.05; paired t test). The error bars represent S.D.
(TIF)
