# Supplementary figures and images for: miR-27b Represses Migration of Mouse MSCs to Burned Margins and Prolongs Wound Repair through Silencing SDF-1a
Source: PLoS One. 2013 Jul 22;8(7):e68972. doi: 10.1371/journal.pone.0068972 (PMC3718818; doi:10.1371/journal.pone.0068972)

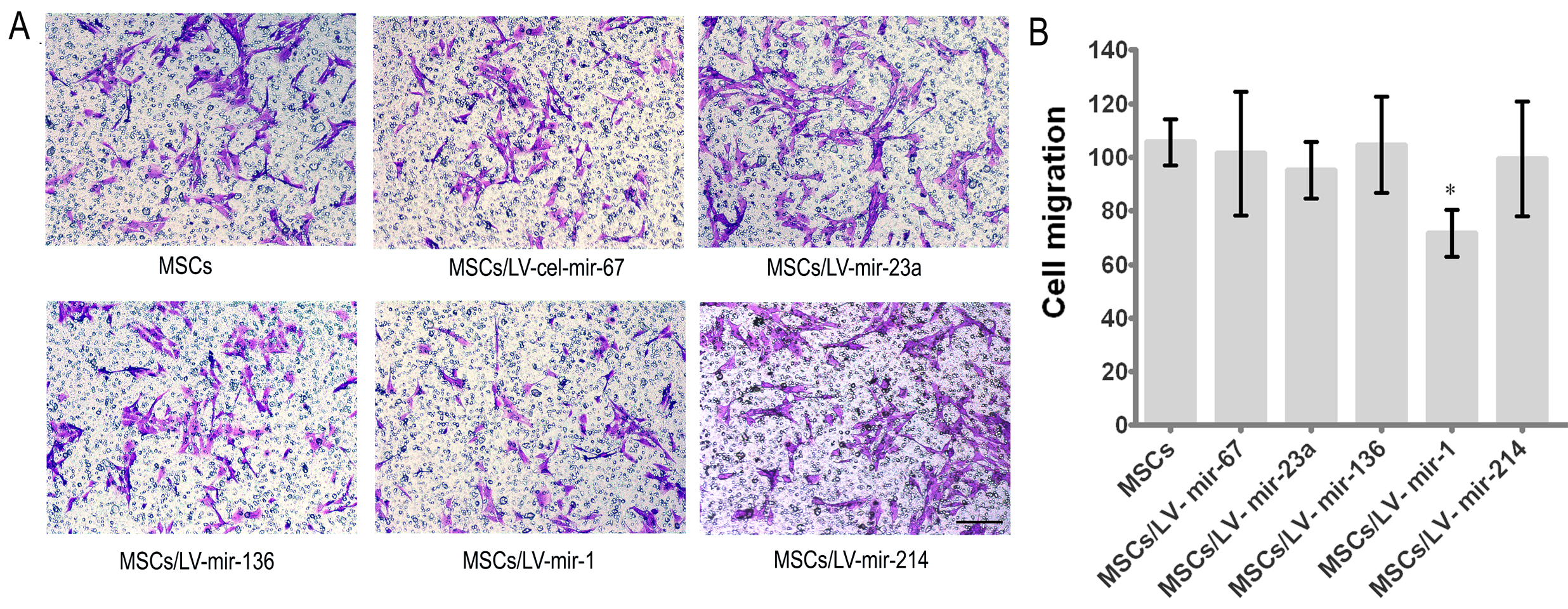

Supplement: Figure S1 — The effects of miR-23a, miR-136, miR-1 and miR-214 on MSC migration. (A) The migration capacity of MSCs over-expressing miR-23a, miR-136, miR-1 or miR-214 in a transwell migration assay was compared to the blank control and negative control. Bar, 100 um (B) Alteration of the chemotactic capacity of MSCs in different niches (n = 3, *p<0.05; independent-sample t test). The error bars represent S.D. (TIF) [file pone.0068972.s001.tif]

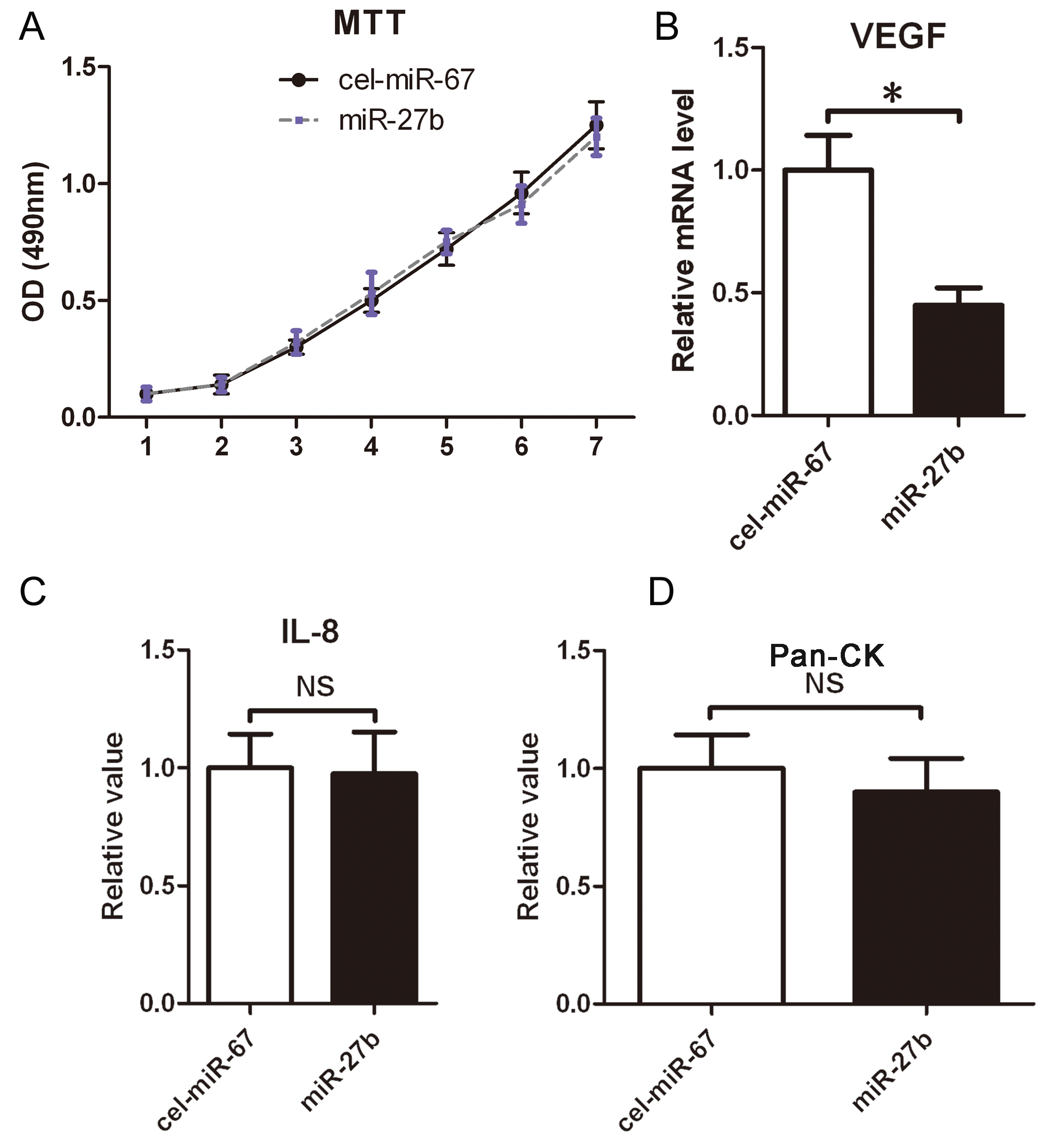

Supplement: Figure S2 — The effects of miR-27b on MSCs behavior. (A) The proliferation of MSCs over-expressing miR-27b in MTT assay was compared to the negative control.(B)The effects of miR-27b on VEGF in qPCR was compared to the negative control.(C) The effects of miR-27b on IL-8 and Pan-CK in ELISA was compared to the negative control.(n = 3, *p<0.05; paired t test). The error bars represent S.D. (TIF) [file pone.0068972.s002.tif]
